# Impact of donor age on short-term outcomes after pediatric split liver transplantation

**DOI:** 10.3389/fped.2023.1131629

**Published:** 2023-04-11

**Authors:** Min Xu, Chong Dong, Chao Sun, Kai Wang, Wei Zhang, Hong Qin, Chao Han, Yang Yang, Fubo Zhang, Zhen Wang, Weiping Zheng, Xinzhe Wei, Wei Gao, Zhongyang Shen

**Affiliations:** ^1^ Department of Pediatric Transplantation, Organ Transplantation Center, Tianjin First Central Hospital, Tianjin, China; ^2^ Tianjin Key Laboratory of Organ Transplantation, Tianjin First Central Hospital, Tianjin, China; ^3^ Organ Transplantation Center, Tianjin First Central Hospital, Tianjin, China

**Keywords:** pediatric split liver transplantation, donor age, short-term outcomes, complications, graft survival rates

## Abstract

**Background:**

Donor shortage is an important limitation of liver transplantation (LT). Split liver transplantation (SLT) may increase the sources of donors and reduce the problem of organ shortage. However, there are no standard criteria of the selection of SLT donor, especially regarding the donor age.

**Methods:**

We retrospectively analyzed the clinical data of children who received initial SLT between January 2015 and December 2021. Based on the age of donors, the patients were divided into groups A (1–10 years old; *n* = 26), B (10–45 years old; *n* = 87), and C (45–55 years old; *n* = 27). The short-term (<1 year after SLT) outcomes of the recipients were analyzed.

**Results:**

A total of 140 patients received SLT from 122 donors. The 1-, 3- and 12-month patient survival rates in group A were 100.0%, and the graft survival rates were 92.3%. The 1-, 3- and 12-month survival rates of patient and graft in group B were 97.7%, 96.6%, and 95.0%, respectively, and in group C were 85.2%, 85.2%, and 81.1%, respectively. The patient survival rate was significantly lower in group C than in groups A and B (*p* = 0.0082). There was no significant difference in graft survival between the three groups (*p* = 0.0545).

**Conclusions:**

Similar results were obtained for pediatric SLT with donors <10 years old and 10–45 years old. Pediatric SLT can be performed with older donors (45–55 years) after strict donor selection and selection of appropriate recipients.

## 1. Introduction

Pediatric patients with end-stage liver disease often require pediatric liver transplantation (LT) (1, 2). However, donor shortage is an important problem that restricts the widespread use of pediatric LT (3, 4). The use of living donor liver transplantation (LDLT) and split liver transplantation (SLT) may increase the sources of donors and reduce the problem of organ shortage (5). In particular, SLT allows a greater number of recipients to benefit from a limited number of donors. Under appropriate conditions, a single liver graft can be shared by two recipients (6, 7), which significantly increases the availability of donors and provides more possibilities for patients on the waiting list.

Many studies have reported that SLT can achieve similar survival results as LDLT and whole liver transplantation (WLT) (1, 8–10). Thus, SLT is safe and effective in pediatric recipients when appropriate donors and recipients are selected. However, there are no global standard criteria of the selection of SLT donor, especially regarding the donor age. For example, Italian guidelines suggest that donors should have an age of 10–50 years, stable hemodynamics, intensive care unit (ICU) stay ≤5 days, transaminase level ≤3 times of normal, and no steatosis on ultrasound scan (11). In contrast, British and Korean guidelines require donors to be younger than 40 years (12, 13). In our country, the consensus proposed that the donor age of SLT should be less than 50 years, stable hemodynamics, intensive care unit (ICU) stay ≤5 days, transaminase level ≤3 times of normal, and total bilirubin level ≤2 times of normal (14). Although the criteria vary between centers, older donors are vulnerable to ischemia-reperfusion injury (15) and graft loss (16, 17). Hence, it is necessary to determine the appropriate age of donors for spitting.

The purpose of this study was to compare the short-term (within 1 year after LT) clinical efficacy and prognosis of different age donors in children with SLT.

## 2. Methods

### 2.1. Study design and participants

**Figure 1:**
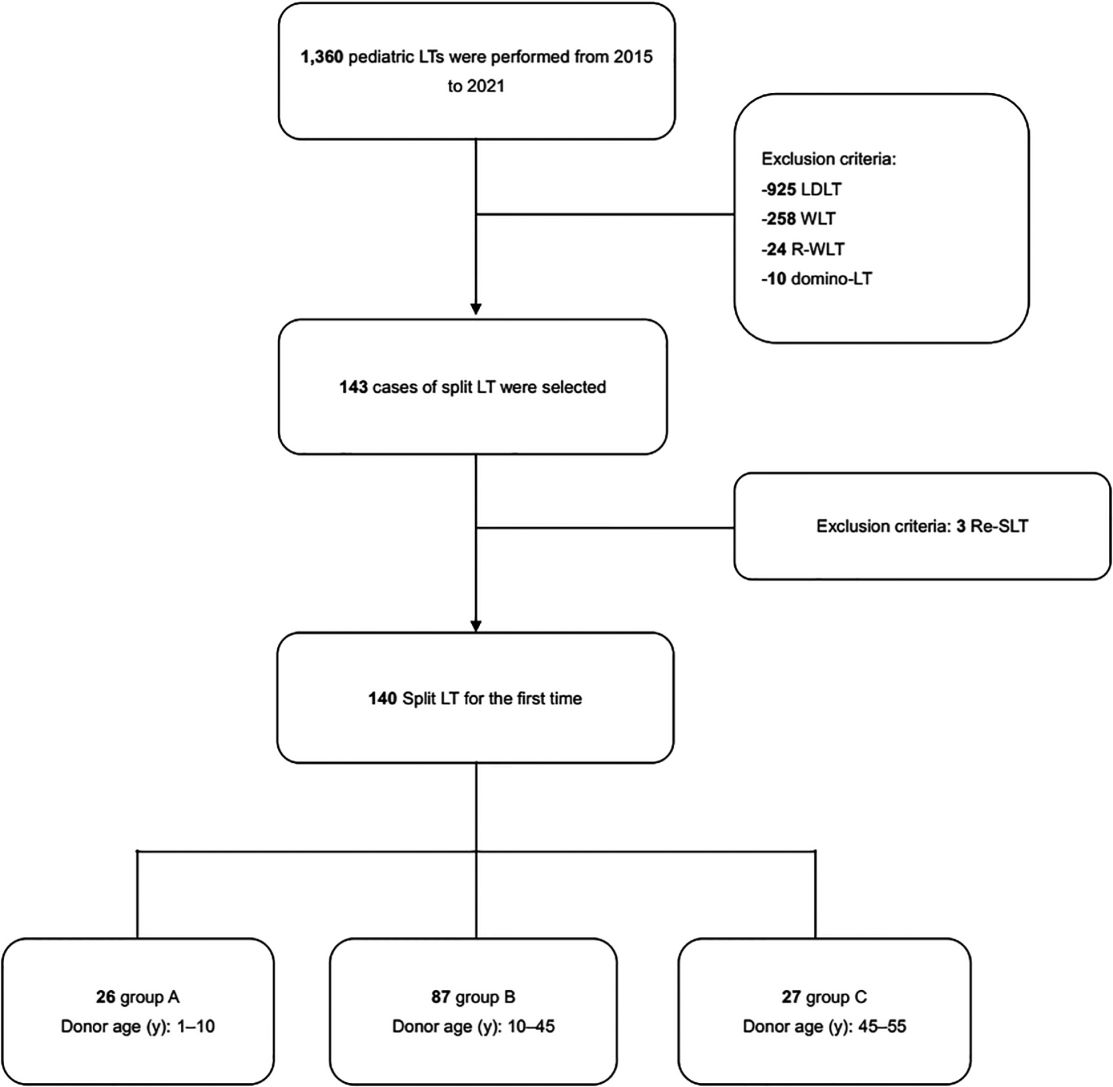
Flowchart showing patient selection.

We retrospectively analyzed the clinical data of pediatric patients who received initial SLT at the Department of Pediatric Transplantation, Tianjin First Central Hospital, between January 2015 and December 2021. The patients were followed up until June 30, 2022. A total of 1,343 recipients underwent 1,360 LTs, among which 925, 258, and 24 cases of LDLT, WLT, and reduced-size LT, respectively, were excluded. We also excluded 10 cases of domino LTs. Among the remaining 143 cases of SLT, 3 cases of re-SLT were excluded. Finally, 140 cases were included in the analysis. We divided the patients into three groups based on the donor age: A (1–10 years), B (10–45 years), and C (45–55 years) (Figure 1). This study was approved by the institutional review board of Tianjin First Central Hospital (approval number: 2023DZX09).

Demographic data of the recipients and donors were compared between the three groups. The short-term (<1 year after SLT) outcomes and complications of the recipients were compared between the groups and the risk factors of graft loss were analyzed.

### 2.2. Surgical procedures

*In situ* splitting refers to splitting the liver parenchyma, followed by reperfusion and cold preservation (3). In comparison, in *ex situ* splitting, perfusion is performed, followed by splitting on the back table. Biopsy were obtained routinely after perfusion with University of Wisconsin (UW) solution or histidine-tryptophan-ketoglutarate (HTK) solution. Macrovesicular steatosis refers to steatosis in which large lipid droplets appear within hepatocytes. Macrovesicular steatosis was graded pathologically: mild <30%, moderate 30%–60%, severe >60% (18).

For younger donors (donor age: 1–10 years), the left lateral segment (LLS) will receive the allocation of the celiac axis or left hepatic artery. Whereas for the portal vein (PV), the trunk was allocated to the extended right lobe (ERL). Similarly, the trunk of the bile duct was allocated to the ERL.

Piggyback LT was performed in all recipients. The portal vein was anastomosed end-to-end with continuous or interrupted sutures. The hepatic artery was sutured with 8-0 or 9-0 non-absorbable sutures under the microscope. Roux-en-Y choledochojejunostomy was performed in all cases.

### 2.3. Definition of early allograft dysfunction (EAD)

The EAD definition proposed by Olthoff et al. (18) was used, which was based on the occurrence of one or more of the following indicators: (1) Total bilirubin (TBIL) ≥10 mg/dl (171 µmol/L) on postoperative day 7 (POD 7); (2) international normalized ratio ≥1.6 on POD7 without anticoagulant use; and (3) alanine aminotransferase (ALT) or aspartate aminotransferase (AST) levels >2,000 U/ml within the first seven postoperative days.

### 2.4. Statistical analysis

We expressed categorical variables as frequencies or percentages, and performed *χ*^2^ test or Fisher's exact probability tests, where appropriate. Shapiro–Wilk test was used to determine whether the measurement variables conformed to a normal distribution, and if so, they were expressed as means ± standard deviation (x ± SD) and analyzed by Student's *t*-test. If the data were not parametric, they were presented as medians (interquartile range, IQR) and compared using the Kruskal–Wallis test. The Kaplan–Meier method was used for survival analysis, and the log-rank test was used to compare the differences in survival distributions between the groups. The multivariable analysis was used to determine the effect of patient and donor variables on graft survival using the Cox's model and Enter method. *p* < 0.05 was considered statistically significant. We analyzed the data using SPSS 20.0 software (IBM Corp., Armonk, NY, USA).

## 3. Results

### 3.1. Overall characteristics

A total of 140 patients received LTs from 122 donors and were divided into three groups: 26 (18.6%), 87 (62.1%), and 27 (19.3%) patients in groups A, B, and C, respectively. The median age of the recipients at the time of transplantation was 8.5 (6.5–30.3) months and the median body weight was 7.6 (6.7–12.0) kg. Cholestatic diseases (116/140, 83.9%) were the most common disease that necessitated LT. The main cause of death of donors in group C was stroke (70.4%), while that in groups A and B was brain trauma (46.7% and 55.0%, respectively). Tables 1, 2 compare the basic characteristics of recipients and donors between the three groups.

**Table 1:** Characteristics of recipient in the three groups.

Recipient characteristics	Group A (*n* = 26)	Group B (*n* = 87)	Group C (*n* = 27)	*p*-value
Age (months)	7.2, 6.0–35.6	9.5, 6.6–31.7	9.0, 7.7–23.4	0.366
Gender (Male/Female)	12/14	39/48	12/15	0.991
Height (cm)	65.0, 63.0–82.5	69.0, 65.0–89.0	70.0, 64.0–82.0	0.447
Weight (kg)	7.2, 6.5–12.1	7.6, 6.7–12.6	8.0, 6.5–11.2	0.767
BMI	16.5, 14.8–17.6	16.0, 14.7–17.8	15.9, 14.7–17.3	0.927
PELD score	15.5, 5.5–20.0	18.0, 7.0–27.0	22.0, 8.0–30.0	0.328
Diagnosis				0.457^a^
Cholestatic diseases	24 (92.3)	67 (77.0)	25 (92.6)	
Metabolic diseases	1 (3.8)	5 (5.7)	1 (3.7)	
Graft failure	0	7 (8.0)	1 (3.7)	
Others	1 (3.8)	8 (9.2)	0	
**Blood type**				
Compatible/Incompatible	21/5	81/6	23/4	0.152^a^

BMI, body mass index; PELD, pediatric end-stage liver disease.

^a^ When the expected value was less than 5, Fisher's method was applied.

**Table 2:** Characteristics of donor in the three groups.

Donor characteristics	Group A (*n* = 15)	Group B (*n* = 80)	Group C (*n* = 27)	*p*-value
Age (years)	5.8, 4.0–7.0	30.5, 21.0–39.0	50.0, 47.1–51.1	–
Gender (Male/Female)	10/5	64/16	23/4	0.374^a^
Weight (kg)	19.0, 15.0–25.0	65.0, 55.0–70.0	70.0, 65.0–75.0	–
BMI	15.1,14.5–16.6	22.9, 20.2–25.1	25.1, 22.5–27.3	–
Cause of death				0.000^a^
Stroke	1 (6.7)	26 (32.5)	19 (70.4)	
Trauma	7 (46.7)	44 (55.0)	8 (29.6)	
Anoxia	5 (33.3)	3 (3.8)	0	
Brain tumor	2 (13.3)	6 (7.5)	0	
Others	0 (0)	1 (1.2)	0	
ALT (U/L)	32.1, 20.0–90.0	26.1, 14.0–63.7	22.0, 14.0–43.0	0.409
AST (U/L)	62.0, 31.8–135.0	34.1, 20.8–71.2	29.0, 18.9–46.5	0.047
TBIL (mmol/L)	11.6, 7.0–21.0	12.2, 7.9–18.6	11.8, 9.3–16.2	0.984
Serum sodium (mmol/L)	149.5, 137.9–159.8	141.3, 138.1–145.6	144.4, 138.7–149.0	0.159

BMI, body mass index; ALT, alanine aminotransferase; AST, aspartate aminotransferase; TBIL, total bilirubin.

^a^ When the expected value was less than 5, Fisher's method was applied.

### 3.2. Details of graft

The left lateral segment (LLS) was the main type of graft used in groups B and C. In contrast, the LLS and extended right lobe (ERL) accounted for half of the grafts each in group A. Meanwhile, in terms of surgical techniques, the grafts in group A were mainly split *ex situ*, while almost all the grafts in groups B and C were split *in situ* (*p* = 0.000). In addition, the organ preservation solution used in groups A and C was mainly the University of Wisconsin (UW) solution, while an equal number of grafts in group B used UW solution and histidine-tryptophan-ketoglutarate (HTK) solution (*p* = 0.003). The median cold ischemia time (CIT) in group A was 520.0 min, which was significantly longer than that in groups B and C (153.0 and 209.0 min, respectively; *p* = 0.000; Table 3). The proportion of steatosis in grafts in group C was 11.1%, which was higher than that in the other two groups; however, there was no significant difference between the three groups (*p* = 0.517). There was no significant difference in the graft-to-recipient weight ratio (GRWR) among the three groups.

**Table 3:** Comparison of characteristics of grafts among the three groups.

*N* = 140	Group A (*n* = 26)	Group B (*n* = 87)	Group C (*n* = 27)	*p*-value
**Graft**				
Type				0.000^a^
LLS	13 (50.0%)	68 (78.2%)	23 (85.2%)	
RLLS	0	11 (12.6%)	4 (14.8%)	
ERL	13 (50.0%)	8 (9.2%)	0	
Surgical techniques				0.000^a^
*In situ*	8 (30.8%)	87 (100.0%)	25 (92.6%)	
*Ex situ*	18 (69.2%)	0	2 (7.4%)	
Weight (g)	228.0, 152.3–370.3	288.0, 250.0–340.0	289.0, 250.0–331.0	0.146
GRWR (%)	2.86 ± 0.992	3.36 ± 1.168	3.63 ± 1.176	0.057
Steatosis (Pathological)				0.517
No	24 (92.3%)	82 (94.3%)	24 (88.9%)	
Mild	2 (7.7%)	5 (5.7%)	3 (11.1%)	
**Fluid preservation**				
UW solution	24 (92.3%)	47 (56.6%)	19 (70.4%)	0.003
HTK solution	2 (7.7%)	36 (43.4%)	8 (29.6%)	

LLS, left lateral segment; RLLS, reduced left lateral segment; ERL, extended right lobe; GRWR, graft-to-recipient weight ratio; UW, university of wisconsin; HTK, histidine-tryptophan-ketoglutarate.

^a^ When the expected value was less than 5, Fisher's method was applied.

### 3.3. Liver function after LT

**Figure 2:**
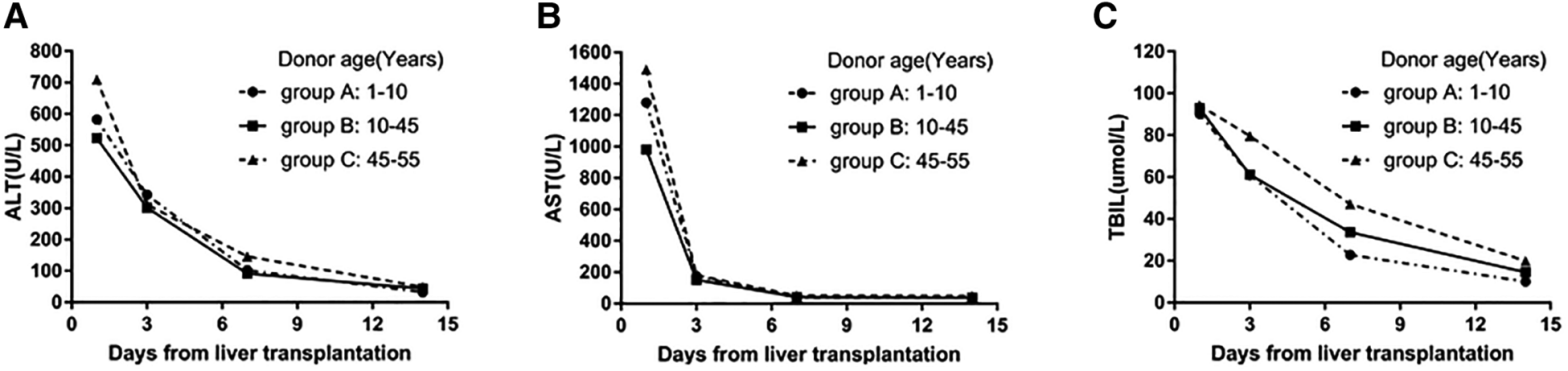
(**A**) Change in the median ALT values on days 1, 3, 7, and 14 after LT among the three groups. (**B**) Change in median AST levels on days 1, 3, 7, and 14 after LT in the three groups. (**C**) The change in median TBIL values on days 1, 3, 7, and 14 after LT in the three groups.

The ALT and AST of the three groups were significantly increased on the first day after LT. The median AST peak level in groups A and C were 1,280.0 (585.9–2,811.3) and 1,490.8 (680.0–2,987.2) U/L, which was not significantly different from that in group B [981.4 (503.7–1,707.0) U/L; *p* = 0.121]. There was no significant difference in the peak ALT level among the three groups [*p* = 0.123; 582.6 (267.7–1093.0), 523.0 (313.3–948.2), and 709.5 (471.8–1582.8) in groups A, B, and C, respectively]. The TBIL was not significantly different between the three groups on days 1, 3, and 14 after LT. However, on day 7 after LT, the TBIL level was higher in group C than that in the other two groups (*p* = 0.035). As shown in Figure 2, the ALT, AST, and TBIL levels in the three groups decreased to normal 14 days after the LT, with no significant difference among the groups (*p* = 0.336, 0.333, and 0.083, respectively).

### 3.4. Complications within 1 year after liver transplantation LT

The incidence of vascular complications was 16.4% (23/140), and that of biliary complications was 8.6% (12/140). The incidence of hepatic artery thrombosis (HAT) in group A was 7.7%, which was higher than that in the other two groups, but there were no significant differences among the three groups (*p* = 0.116). The incidence of hepatic vein stricture and biliary leakage was highest in group B (10.3% and 8.0%, respectively), but was not significantly different compared with the other two groups (*p* = 0.483 and 0.404, respectively). Overall, the incidence of EAD was 28.9% (39/140), with no significant difference among the three groups (*p* = 0.111). The complications in each group are shown in Table 4.

**Table 4:** Complications after liver transplantation.

Complication	Group A (*n* = 26)	Group B (*n* = 87)	Group C (*n* = 27)	*p*-value
HAT	2 (7.7%)	1 (1.1%)	0	0.116
PVT	1^a^ (3.8%)	0	1 (3.7%)	0.142
Portal vein stricture	1 (3.8%)	4 (4.6%)	2 (2.4%)	0.856
Hepatic vein stricture	1 (3.8%)	9 (10.3%)	1 (3.7%)	0.483
Biliary leakage	0	7^b^ (8.0%)	1 (3.7%)	0.404
Biliary stricture	2 (7.7%)	1 (1.1%)	1 (3.7%)	0.438
EAD	9 (34.6%)	19 (21.8%)	11 (40.7%)	0.111

HAT, hepatic artery thrombosis; PVT, portal vein thrombosis; EAD, early allograft dysfunction.

^a^ One patient had both hepatic artery thrombosis and portal vein thrombosis.

^b^ One patient had both biliary leakage and stricture.

### 3.5. Causes of graft loss and recipient death within 1 year after LT

A total of 3 recipients underwent retransplantation, including 2 in group A and 1 in group C (Table 5). The main causes of retransplantation were HAT and portal vein thrombosis (PVT). In group A, one patient had graft loss due to HAT and PVT, and another had HAT. Both patients underwent retransplantation and survived. In group C, one recipient underwent retransplantation due to PVT and graft dysfunction, and finally died of graft failure 1 month after the operation.

**Table 5:** Reasons for retransplantation.

Retransplantation (*N* = 3)	Cause of loss	Date of loss (post transplantation)
Group A (*n* = 2)	HAT and PVT × 1	2 days
	HAT × 1	2 days
Group B (*n* = 0)	–	–
Group C (*n* = 1)	PVT × 1	14 days

HAT, hepatic artery thrombosis; PVT, portal vein thrombosis.

In this study, there were 9 cases of recipient death: none in group A, 4 (4/87, 4.6%) in group B, and 5 (5/27, 18.5%) in group C (Table 6). The causes of death included graft failure, accident, chronic rejection, interstitial pneumonia, and sepsis. Four patients died within 30 days after LT, including 1 case of graft failure, 1 case of interstitial pneumonia, and 2 cases of sepsis.

**Table 6:** Causes of recipient death and postoperative time after LT.

Recipient death (*N* = 9)	Cause of death	Date of death (after transplantation)
Group A (*n* = 0)	–	–
Group B (*n* = 4)	Sepsis ×1	9 days
	Graft failure ×1	46 days
	Interstitial pneumonia ×1	2 months
	Chronic rejection ×1	12 months
Group C (*n* = 5)	Sepsis ×1	9 days
	Graft failure ×2	34 days, 14 days
	Interstitial pneumonia ×1	22 days
	Accident ×1	7 months

### 3.6. Survival of pediatric recipients and grafts

**Figure 3:**
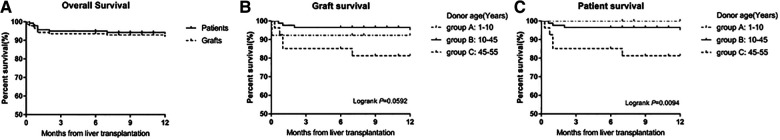
(**A**) Survival of patients and grafts for 140 children who underwent liver transplantation. (**B**) Survival of grafts in the three groups. There were no significant differences in the graft survival rates among the three groups (*p* = 0.0592). (**C**) Survival rates of the recipients in the three groups. The patient survival rates were significantly lower in group C than in groups A and B (*p* = 0.0094).

As shown in Figure 3A, the 1-, 3- and 12-month overall patient survival rates were 95.7%, 95.0%, and 93.5%, respectively; and the 1-, 3- and 12-month overall graft survival rates were 94.3%, 93.6%, and 92.0%, respectively. The 1-, 3- and 12-month patient survival rates in group A were all 100.0%, and graft survival rates were all 92.3%. The 1-, 3- and 12-month patient survival rates in group B were 97.7%, 96.6% and 95.2%, respectively, and the graft survival rates were similar. And the The 1-, 3- and 12-month survival rates for both patients and grafts in group C were 85.2%, 85.2%, and 81.3%, respectively. The patient survival rate was significantly lower in group C than in groups A and B (*p* = 0.0094). There was no significant difference in graft survival between the three groups (*p* = 0.0592).

### 3.7. Risk factors of graft loss within 1 year after LT

In the univariate analysis, increased blood loss significantly increased the risk of graft loss (*p* = 0.031; Table 7). However, PVT was a significant factor affecting graft survival (*p* = 0.015).

**Table 7:** Predictors of 1-year graft survival rate in cox analysis.

Variable		Univariate		Multivariate	
		*p*	HR	95% CI	*p*
**Recipient**					
Age (months)	8.5 (6.5–30.3)	0.868			
Gender	Male (*n* = 63)	0.769	** **	** **	** **
	Female (*n* = 77)				
Weight (kg)	7.6 (6.7–12.0)	0.587			
BMI	16.0 (14.7–17.6)	0.079	0.656	0.392–1.100	0.110
PELD	18.0 (7.3–26.8)	0.526			
Preoperative AST (U/L)	199.2 (104.4–346.1)	0.506			
Preoperative ALT (U/L)	126.5 (68.7–213.5)	0.928			
Preoperative TBIL (mmol/L)	340.4 (62.8–356.9)	0.103	1.003	1.000–1.007	0.060
**Donor**					
Age (Years)	Group A (*n* = 26)	0.055	0.378	0.015–9.353	0.552
	Group B (*n* = 87)		1		
	Group C (*n* = 27)		4.429	1.067–18.385	0.040
Weight (kg)	65.0 (45.0–70.0)	0.367			
Preoperative AST (U/L)	35.2 (20.9–73.4)	0.268			
Preoperative ALT (U/L)	29.0 (15.2–60.0)	0.542			
Preoperative TBIL (mmol/L)	12.0 (8.1–18.2)	0.339			
Preoperative Na (mmol/L)	141.4 (138.2–148.7)	0.993			
**Graft**					
Type	LLS (*n* = 104)	0.962			
	RLLS (*n* = 15)				
	ERL (*n* = 21)				
Surgical techniques	*In situ* (*n* = 120)	0.677			
	*Ex situ* (*n* = 20)				
Weight (g)	287.5 (240.0–343.8)	0.733			
GRWR (%)	3.32 ± 1.16	0.831			
Steatosis (Pathological)	No (*n* = 130)	0.573			
	Mild (*n* = 10)				
Fluid preservation	UW (*n* = 90)	0.325			
	HTK (*n* = 46)				
**Intraoperative**					
Operation duration (min)	495.0 (415.0–555.0)	0.258			
CIT (min)	185.5 (116.3–461.3)	0.688			
Anhepatic phase (min)	48.0 (41.3–59.0)	0.216			
Blood loss (mL)	400.0 (212.0–600.0)	0.031	1.002	0.999–1.005	0.192
Blood transfusion (U)	3.0 (2.0–4.0)	0.094	0.773	0.427–1.398	0.394
Plasma transfusion (mL)	200.0 (200.0–430.0)	0.527			
**Postoperative**					
ICU stay (d)	3.0 (2.0–4.0)	0.217			
HAT	No (*n* = 137)	0.069	1	** **	** **
	Yes (*n* = 3)		14.306	0.605–338.291	0.099
Portal vein stenosis	No (*n* = 133)	0.491			
	Yes (*n* = 7)				
Portal vein thrombosis	No (*n* = 138)	0.431	1		
	Yes (*n* = 2)	0.015	20.555	1.390–304.019	0.028
Hepatic vein stricture	No (*n* = 129)	0.861			
	Yes (*n* = 11)				
Biliary complications	No (*n* = 129)	0.122	1		
	Yes (*n* = 11)		0.892	0.079–18.440	0.991
EAD	No (*n* = 101)	0.598			
	Yes (*n* = 39)				

Factors with *p*-value <0.2 in the univariate analysis were included in the multivariate analysis. Table 7 shows the risk factors affecting graft survival in the Cox multivariable analysis. The results showed that donor age >45 years [hazard ratio (HR) = 4.429, *p* = 0.040] and postoperative PVT (HR = 20.555, *p* = 0.028) were independent risk factors of graft loss.

## 4. Discussion

SLT significantly alleviates the problem of organ shortage (6). The influence of donor age on the recipient outcomes is unclear, and there are no clear guidelines regarding donor age (19). Some studies have shown that donors aged 60–80 years can safely donate their liver with good results; however, these studies were conducted in WLT (20, 21). There is no unified standard for splitting, especially with regard to the donor age. Donor age significantly affects the survival and prognosis of grafts (12, 17), but there is no clear standard for splitting (12). In this study, we compared the early clinical outcomes and prognosis of SLT with donors of different ages to evaluate the splitability according to the different ages of donors.

In this study, the peak ALT and AST levels were significantly increased in groups A and C after LT, although there was no significant difference compared with the level in group B. This may be related to the significantly prolonged CIT in group A, which results in hepatic ischemic injury. However, although the CIT was shorter in group C than in group A, the vulnerability to ischemic injury caused by the older donors in group C may explain the significantly increased AST level (22). In addition, the transaminase levels in each group decreased to the normal value after 2 weeks, with no significant difference between the groups. Furthermore, there was no difference in the recovery of liver function between the three groups. However, the decrease in TBIL in group C was slower than that in groups A and B. The TBIL level on day 7 after the operation was significantly higher in group C than that in the other groups, which may be due to the delayed recovery of liver function in cases of older donors.

In terms of postoperative complications, group A had the highest incidence of HAT within 1 year after LT, although there was no statistical difference when compared with the other two groups. The two recipients who developed HAT in group A were small body weight recipients (5.3 kg and 5 kg). The thrombosis in these two patients with small body weight may be caused by small vessel diameter or vessel mismatch (23–26). Therefore, anastomosis of the hepatic artery must be performed carefully, preferably with use of a microscope. Some studies have shown that SLT can increase the incidence of biliary complications (27, 28). However, in this study, the incidence of biliary complications (including bile leakage and biliary stricture) within 1 year after operation was 8.6% (12/140), which was lower than the 13.6% reported by the Society of Pediatric Liver Transplantation (29). In the present study, the incidence of EAD was highest in group C and lowest in group B, but there was no significant difference between the three groups. In a previous study, Olthoff et al. (18) concluded that donor age older than 45 years significantly increased the risk of developing EAD (RR = 1.94, 95% CI: 1.21, 3.92), and they also confirmed that EAD increased the risk of graft loss (RR = 7.4, 95% CI: 3.4, 16.3). Unlike the results of the aforementioned study, in the present study, the EAD was not a risk factor for graft survival. Furthermore, other research has suggested that donor age, allograft steatosis, donor liver mass, and DCD (donation after cardiac death) status are associated with the development of EAD (30).

The 1-year survival rate of the recipients in group A was 100%. Moreover, even though the median CIT was nearly 9 h in group A, these excellent results were still achieved, and the 1-year graft survival rate was over 90%. Although there is still some controversy surrounding SLT using younger donors (<10 years), our study shows good results with this subset of donors. It follows that even SLT using younger age donors can lead to good outcomes with use of improved surgical techniques as well as refined postoperative care. These findings differ from studies that show that prolonged CIT may significantly increase the risk of graft loss (12, 27, 31, 32). Recent data from Europe show that increasing the CIT from 6 to 10 h corresponds to an HR of 1.33 (12). Similarly, Lozanovski et al. reported that the influence of CIT shows a difference when comparing between ischemia time <10 h and ≥10 h (32). In the Cox univariate analysis in the present study, CIT was not the main factor affecting the survival of recipients. However, our previous studies have shown that prolonged CIT can lead to poor prognosis in recipients of WLT. In univariate and multivariate analysis, graft type, hepatic artery thrombosis, portal vein stenosis, hepatic vein stenosis and biliary complications were not the risk factors affecting the short-term survival of the graft. Actually, however, it is well known that HAT can negatively affect graft survival. That may be explained by the small sample size of this study. In contrast, in the multivariate analysis of the present study, donor age >45 years (HR = 4.429) and postoperative PVT (HR = 25.550) were the main risks of graft loss.

Previously, we have reported that donors younger than 7 years old have achieved good results in SLT, which shows that carefully selected pediatric donors can be safely and effectively used in SLT in children (33). In this study, we included recipients who received donors aged 1–55 years, of which 26 recipients received pediatric donors aged 1–10 years. The 26 recipients received the LLS and ERL of 15 donors, and good results were achieved. Nevertheless, pediatric donors still have many risk factors, such as a high incidence of HAT, which necessitates paying attention to protecting the hepatic artery of the donor and recipient in the process of splitting the liver, as well as closely monitoring the blood flow of the hepatic artery after surgery. In addition, for younger donors, according to the previous experience of our center, the weight of the donor selected for the split should be >15 kg, which can ensure the recipient has a good prognosis.

The donors we previously selected for SLT were younger than 45 years old. Recently, however, due to the shortage of donors, we began to expand the age selection criteria of donors. In this study, the oldest donor we selected was 55 years old, which is higher than the standard of most centers. In the present study, the survival rate of donors older than 45 years was the lowest; however, this rate does not impact the use of this age group of donors for splitting. First, the number of these donors is small and vulnerable to interference. Second, we performed SLT in 14 cases with donors older than 45 years in 2021, and achieved good short-term survival (no graft loss or recipient death) and few postoperative complications, indicating that older donors may not be the limiting factor in splitting. However, the follow-up time in this study was 1 year; hence, it does not reflect the long-term outcome of the recipients. According to the experience of our center, donor candidates older than 45 years are suitable for splitting, provided there is reasonable selection of donors and recipients; that is, for example, the donor has no hypotension before operation, no or little use of pressor drugs, ALT and AST are less than three times the upper limit of normal, ICU stay time is less than 5 days, and there is no evidence of systemic infection and no moderate-to-severe fatty lesions. Severely ill recipients should not be selected. Furthermore, the choice of *in situ* splitting can considerably reduce the damage to vessels and bile ducts and bleeding after reperfusion. Careful postoperative care and regular monitoring are essential. These measures may reduce the risk of graft loss and reduce the incidence of complications.

However, this study has several limitations. First, this is a single center retrospective study with a limited sample size. Second, we only reported the short-term outcomes for the recipients and lacked the results of long-term follow-up. Finally, we reported that 140 recipients received 122 donors due to the fact that there was a part of the graft that was allocated to other centers. And adult recipients were not included in the study. Therefore, no information was available for these recipients, which also leads to mismatches in the number of donors and recipients.

## 5. Conclusion

In this study, the application of SLT in pediatrics with donor age <10 years resulted in similar satisfactory outcomes compared with those of donor age 10–45 years. For the 45–55-year-old donor age group, although patient survival was reduced, both the patient and graft 1-year survival rate exceeded 80%. Under the conditions that donors are selected according to strict criteria and suitable recipients are selected, this subset of donors can also be applied to pediatric SLT. However, the results of long-term follow-up require further study.

## Data Availability

The original contributions presented in the study are included in the article/Supplementary Material, further inquiries can be directed to the corresponding author.
